# Role of the Checkpoint Clamp in DNA Damage Response

**DOI:** 10.3390/biom3010075

**Published:** 2013-01-16

**Authors:** Mihoko Kai

**Affiliations:** Department of Radiation Oncology, Johns Hopkins University, School of Medicine, Baltimore MD 21231, USA; E-Mail: mkai2@jhmi.edu; Tel.: +1-410-614-9223

**Keywords:** DNA damage checkpoint, checkpoint clamp, DNA repair, Rad9

## Abstract

DNA damage occurs during DNA replication, spontaneous chemical reactions, and assaults by external or metabolism-derived agents. Therefore, all living cells must constantly contend with DNA damage. Cells protect themselves from these genotoxic stresses by activating the DNA damage checkpoint and DNA repair pathways. Coordination of these pathways requires tight regulation in order to prevent genomic instability. The checkpoint clamp complex consists of Rad9, Rad1 and Hus1 proteins, and is often called the 9-1-1 complex. This PCNA (proliferating cell nuclear antigen)-like donut-shaped protein complex is a checkpoint sensor protein that is recruited to DNA damage sites during the early stage of the response, and is required for checkpoint activation. As PCNA is required for multiple pathways of DNA metabolism, the checkpoint clamp has also been implicated in direct roles in DNA repair, as well as in coordination of the pathways. Here we discuss roles of the checkpoint clamp in DNA damage response (DDR).

## 1. Introduction

The homologs of Rad9, Rad1 and Hus1 in fission yeast were originally discovered by genetic screens to identify genes that affect cell survival upon genotoxic stresses. On the basis of computational modeling, electron microscopy (EM), and biochemical analyses, it is known that Rad9, Rad1 and Hus1 form a heterotrimeric ring structure that resembles PCNA [1,2,3,4,5]. The crystal structure of the checkpoint clamp complex was determined in 2009, and the structure indeed shows a closed-ring architecture of the checkpoint clamp, formed through a head-to-tail association of its subunits similar to that in PCNA [6,7,8]. The homotrimeric complex PCNA is recruited to DNA by the replication clamp loader RFC1–5 complex, and stimulates DNA polymerases and other enzymes for DNA repair [9,10,11,12,13,14,15,16,17,18,19]. Analogously, the checkpoint clamp is recruited to DNA damage sites by the checkpoint clamp loader complex that consists of Rad17 and RFC 2–5 [2]. Although the PCNA and the checkpoint clamp function in different pathways of DNA metabolism, several DNA repair enzymes interact with both clamps. One big difference between PCNA and the checkpoint clamp is that the checkpoint clamp contains an unstructured *C*-terminal tail on Rad9. This tail is required for checkpoint activation through interaction with TopBP1 [20,21,22]. We are beginning to understand mechanisms of the checkpoint activation through the checkpoint clamp complex. However, roles of the checkpoint clamp in DNA repair regulation are largely unknown. This review describes current understanding of checkpoint activation and repair regulations by the checkpoint clamp. 

## 2. Role of the Checkpoint Clamp in Checkpoint Activation

Depending on the nature of genomic perturbation, different components of the checkpoint factors are employed to deal with DNA damage. The phosphoinositide kinase-related kinases, ATM and ATR, operate near the apex of checkpoint pathways [20,23]. ATM-dependent pathways are initiated primarily by double-strand breaks (DSBs), while ATR responds to a broad spectrum of DNA damage and replication disruption, especially during the S-phase. Critical functions of ATM and ATR involve activation of the downstream checkpoint effector kinases Chk2 and Chk1, respectively. These downstream effector kinases regulate cell cycle progression by phosphorylating cell cycle proteins such as Cdc25 [24]. 

It has been thought that the checkpoint clamp complex functions in the ATR-dependent pathway, because it is required for activation of Chk1 but not for Chk2 [25,26,27]. The role of the checkpoint clamp in Chk1 activation is to bind TopBP1, which stimulates ATR-mediated Chk1 phosphorylation via TopBP1’s activation domain (AD), a domain that binds and activates ATR [20,21,22]. Similar results were obtained by studies of a budding yeast homolog of TopBP1, Dpb11 [28,29]. Interaction between the checkpoint clamp and TopBP1 requires the *C*-terminal tail of Rad9. Human Rad9 contains several phosphorylation sites, most of which are constitutively phosphorylated throughout the cell cycle [22]. This interaction is indeed required for Chk1 activation, because “tailless” Rad9 fused with TopBP1 is able to activate Chk1. Importantly, this complex must be recruited to DNA by the Rad17-RFC checkpoint-clamp loader for Chk1 activation [22]. The *C*-terminal tail, however, is not required for clamp formation or clamp loading [6,7]. Interestingly, two distinct mechanisms of ATR activation through the checkpoint clamp are proposed in budding yeast. Biochemical studies identified two factors, the checkpoint clamp and the Dpb11/TopBP1 replication protein, as potential activators of Mec1/ATR. The author showed that G1 phase activation of Mec1/ATR is achieved by the Ddc1/Rad9 subunit of the checkpoint clamp, while Dpb11/TopBP1 is dispensable. In G2, however, the checkpoint clamp activates Mec1/ATR by two distinct mechanisms. One mechanism involves direct activation of Mec1/ATR by Ddc1/Rad9, while the other proceeds by Dpb11/TopBP1 recruitment mediated through Ddc1/Rad9 phosphorylation [30]. 

Activation of the checkpoint requires recruitment of the checkpoint clamp to chromatin by the checkpoint clamp loader upon replication stresses. Unlike the PCNA homotrimeric complex that three identical subunits of PCNA impart symmetry to its structure and provide the same interaction surface to the clamp loader RFC, the checkpoint heterotrimeric clamp complex loading requires stricter control (because the interaction sites with RFC should be particular sites of one of the checkpoint clamp components). Rad17 has been shown to interact directly with Rad1 and Rad9 [2,8,31], implicating these two subunits in clamp opening. In fact, it has been suggested that Rad1–Rad9 interface is the weakest and the most likely opening site as evidenced by computational analysis, as well as an examination of interface buried surface areas [6,8]. Further studies are needed to determine the mechanism of the checkpoint clamp opening that is required for the clamp loading and also unloading. 

## 3. Role of the Checkpoint Clamp in DSB Repair

Biochemical analyses have shown that the checkpoint clamp preferentially binds to 5’ recessed DNA [32], and single-strand DNA areas on double-strand DNA seem to be required for checkpoint activation [20]. These 5’ recessed structures could be generated in many biological processes in response to many types of genotoxic stresses, and the checkpoint clamp is recruited to chromatin in response to these stresses, including DNA replication inhibition, ultraviolet light, alkylation, and ionizing radiation (IR) [32]. Rad9^−/−^ and Rad9 knockdown cells are sensitive to these genotoxic treatments [27,33]. Therefore, the checkpoint clamp plays a role in response to DSBs, as well as to replication perturbation. Interestingly, however, Rad9^−/−^ cells are not defective in the Chk2 phosphorylation that is activated in response to DSBs. Furthermore, the *C*-terminal tail is not required for resistance to IR [27], implying that the “tailless” clamp might play a direct role in DSB repair. Indeed, the checkpoint clamp proteins are recruited to DSB sites, and the foci co-localize with γH2AX foci [34]. 

The sequence of the DDR protein-loading to chromatin has been shown in response to DSBs in yeast. The MRE11 and the ATM-related Tel1 kinase are the first proteins detected at DSBs. Next, the replication protein A (RPA) single-strand DNA (ssDNA) binding protein relocalizes to resected ssDNA areas and recruits checkpoint proteins. Later, and only in the S and G2 phase, the homologous recombination proteins assemble at the site. Although the checkpoint clamp loader and clamp are recruited to the site before the homologous recombination proteins, they are not required for the recruitment of a homologous recombination protein Rad52 [35]. It is also shown in mammalian cells that ATM and MRE11 nuclease generate RPA-coated ssDNA, and ATR/ATRIP are subsequently recruited to the sites. MRE11 is required for ATM activation [36,37,38,39], and RPA coated ssDNA is required for loading of the checkpoint clamp and ATRIP [40,41]. CtIP is recruited to the DSB sites in an MRN (MRE11-RAD50-NBS1)-complex and ATM-activation-dependent manner, and is required for DSB-end resection. However, the timing of the CtIP recruitment is significantly delayed compared to that of NBS1 [42]. The CtIP protein interacts with BRCA1 protein, and the BRCA1-CtIP complex is thought to mediate 5’ end resection of DSBs, which is abrogated by three independent tumor-associated mutations in the BRCT domain of BRCA1 [43,44]. A CtIP mutant that fails to interact with BRCA1 was shown to have a significant defect in HR in chicken DT40 cells [45]. However, another group showed that the same CtIP mutant is competent in HR, and that BRCA1 and CtIP might play distinct roles in DSB repair pathways by genetic analyses [46]. Further studies are required to verify functions of BRCA1 and CtIP in the HR process. 

A two-step model has been proposed for DSB-end resection. In this model, the MRN complex and CtIP initiate the resection process to remove nucleotides. After this first step, the EXO1 exonuclease and BLM helicase (a RecQ-like helicase)-DNA2 helicase/endonuclease function redundantly to carry out further generation of long 3’ ssDNA tails [33,47,48,49,50,51,52]. Another RecQ-like helicase WRN is also shown to be involved in the DSB resection process in the Xenopus egg extract system [53,54]. However, the WRN protein fails to support reconstitution of DSB resection with human proteins *in vitro* [55]. It has been shown that the loading of a checkpoint clamp component Rad9 requires resection of DSB ends by CtIP [56]. However, the precise timing of the checkpoint clamp loading to chromatin upon damage remains elusive in mammalian cells.

The author’s group has shown that Rad9^Ser272^ becomes phosphorylated during unperturbed S-G2 phase as well, and this phosphorylation occurs in an ATM-dependent manner. Our data indicate that Rad9^Ser272^ phosphorylation governs repair pathways [57]. ATM-dependent Rad9^Ser272^ phosphorylation is not required for survival or checkpoint activation after DNA damage. This phenotype is reminiscent of BRCA1-S988 cells. BRCA1-deficient human cells expressing BRCA1-S988A have defects in homologous recombination (HR). However, the mutant cells retain normal checkpoint function and are resistant to IR, implying that the HR function of BRCA1 is distinct from its other functions in the DNA damage response [58]. Importantly, knock-in mice expressing an S971A mutant (human S988) develop mammary and endometrial tumors after treatment with DNA-damaging agents [59].

This Rad9 phosphorylation requires the MRN complex, confirming requirement of ATM kinase activation for Rad9^Ser272^ phosphorylation. Furthermore, the mutant cells show a defect in homologous recombination (HR), and induce GCRs. Interestingly GCRs are suppressed in a p53^−/−^ background. These results indicate that the checkpoint clamp is involved in HR repair regulation. Further investigation is required to define the function of the checkpoint clamp and ATM-dependent phosphorylation of Rad9 in control of DSB repair. 

## 4. Role of the Checkpoint Clamp in Other Repairs

Biochemical and genetic studies have revealed functional interactions between the checkpoint clamp and repair proteins that include translesion polymerases and base excision repair enzymes [60,61]. 

A checkpoint clamp-loader component Rad17 is required for replication perturbation-induced mutagenesis by translation polymerases, polymerase κ and ζ, in fission yeast. Checkpoint activation is required for transcriptional induction of polymerase κ, and the checkpoint clamp components Hus1 and Rad1 physically interact with polymerase κ [60,61]. Similar results were obtained in budding yeast. The checkpoint clamp physically interacts with polymerase ζ, and is partially required for pol ζ-dependent mutagenesis [62]. 

Biochemical analyses have shown interaction between the checkpoint clamp and the long-patch base excision repair machinery, MutY DNA glycosylase, DNA polymerase β, and Flap endonuclease I. The checkpoint clamp complex enhanced enzymatic activities of these proteins [63,64,65,66]. Nevertheless, confirmation of the biochemical results at the cellular level is required. Some of these repair proteins, including translesion polymerases and Flap endonuclease I, have also been shown to interact with PCNA, and PCNA can stimulate the long-patch base excision repair, as well as translesion synthesis [67,68,69]. Why are two clamps needed for repair pathways? This is still an important, yet unanswered, question.

**Figure 1:**
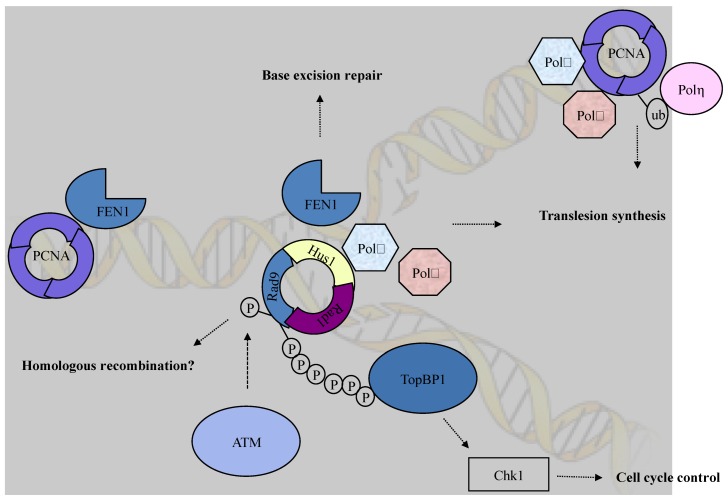
The checkpoint clamp (Rad9-Husl-Radl) complex plays various roles in DNA damage response pathways. The *C*-terminal tail of Rad9 is required for Chk1 activation through interaction with TopBP1. In addition to the role in checkpoint activation, the checkpoint clamp functions in multiple DNA repair pathways such as translesion synthesis and base excision repair.

## 5. Conclusions

Since it was realized that Rad9, Rad1 and Hus1 form a PCNA-like ring structure, significant progress has been made in understanding the function of the checkpoint clamp. Despite much progress, however, the function of the checkpoint clamp, especially in regulation of DNA repair pathways, remains largely unknown. 
